# Spider web and silk performance landscapes across nutrient space

**DOI:** 10.1038/srep26383

**Published:** 2016-05-24

**Authors:** Sean J. Blamires, Yi-Hsuan Tseng, Chung-Lin Wu, Søren Toft, David Raubenheimer, I.-Min Tso

**Affiliations:** 1 Department of Life Science, Tunghai University, Taichung 40704, Taiwan; 2 Evolution & Ecology Research Centre, School of Biological, Earth & Environmental Sciences, The University of New South Wales, Sydney 2052, Australia; 3 Department of Life Science, National Chung-Hsing University, Taichung 40227, Taiwan; 4 Center for Measurement Standards, Industrial Technology Research Institute, Hsinchu 30011, Taiwan; 5 Department of BioScience, Building 1540, Aarhus University, Ny Munkegade 116, DK-Aarhus 8000 C, Denmark; 6 The Charles Perkins Centre, Faculty of Veterinary Science & School of Biological Sciences, The University of Sydney, Sydney NSW 2006, Australia

## Abstract

Predators have been shown to alter their foraging as a regulatory response to recent feeding history, but it remains unknown whether trap building predators modulate their traps similarly as a regulatory strategy. Here we fed the orb web spider *Nephila pilipes* either live crickets, dead crickets with webs stimulated by flies, or dead crickets without web stimulation, over 21 days to enforce spiders to differentially extract nutrients from a single prey source. In addition to the nutrients extracted we measured web architectures, silk tensile properties, silk amino acid compositions, and web tension after each feeding round. We then plotted web and silk “performance landscapes” across nutrient space. The landscapes had multiple peaks and troughs for each web and silk performance parameter. The findings suggest that *N. pilipes* plastically adjusts the chemical and physical properties of their web and silk in accordance with its nutritional history. Our study expands the application of the geometric framework foraging model to include a type of predatory trap. Whether it can be applied to other predatory traps requires further testing.

Predators have been demonstrated to use flexible foraging behaviours to balance their nutrient gains when food quality varies temporally or spatially^1^,^2^,^3^,^4^. Depending on its sensory modalities, a predator may use an assortment of cues to assess the nutritional value of different prey before deploying an appropriate behavioural response^5^,^6^,^7^.

The geometric framework model estimates the homeostatic decisions of animals in relation to their simultaneous exposure to multiple nutrients^8^,^9^. The model is constructed by building a Cartesian space, called nutrient space, with axes representing two or more nutrients of interest^8^,^9^,^10^,^11^. Within this space foods are plotted as rails projecting from the origin at an angle representing the ratio (or balance) of the nutrients they contain. As the animal eats, it’s nutritional state changes along the same trajectory representing the food it is eating. Its current nutritional state can be represented by an intake point, whose position in the space is determined by which, and how much, food(s) it has eaten. This model enables the nutritional states of animals to be manipulated in multiple dimensions to achieve a spread of points across the nutrient space^8^.

Under the geometric framework an animal’s nutrient intake can be related to fitness parameters such as longevity or fecundity by constructing a response surface representing the effects of the amounts or ratios of the nutrients eaten against a measure of fitness (the “fitness landscape”)^9^,^10^,^12^. Terms associated with geographical landscapes are often used to describe the patterns observed in fitness landscapes. For instance the landscapes are sometimes said to have “peaks” or “summits” and “troughs” or “valleys” and rise or fall along “contours”^9^. Where fitness parameters (e.g. longevity or fecundity) cannot be directly plotted, surrogate parameters are collapsed down to a single parameter to plot the landscapes. Generally, such landscapes are called “performance landscapes” as they are not necessarily associated with any fitness costs or benefits, although they may not be associated with any aspect of “performance” either^9^. When a fitness or performance landscape peaks at a single location in nutrient space the location of the peak is considered the performance maxima for the trait plotted^1^,^3^,^9^,^10^. The presence of multiple peaks and troughs in a performance landscape indicate that there is multiple performance maxima for the trait^11^,^12^,^13^. The framework has now been used to explain the foraging decisions made by predatory beetles^1^,^3^, ants^14^ and cursorial spiders^1^,^2^,^15^.

Stationary predators that build traps, such as spiders, caddisfly, ant lions and glow worms, encounter an unpredictable range of prey^16^,^17^,^18^,^19^,^20^. There is evidence that the intake of certain nutrients, in particular protein and lipid, are critically important for trap building predators to regulate. For instance, growth, egg production, silk production and functional stoichiometry in the orb web spiders *Argiope* spp. are associated with variations in lipid and protein uptake^21^,^22^,^23^. Furthermore, protein consumption has been found to correlate strongly with variations in spider web architecture and the physico-chemical properties of the constituent silks^24^,^25^,^26^. Nonetheless, knowing that predators vary the architecture and/or the chemical and physical properties of their traps or trap materials in response to the different types of prey encountered tells us nothing about the underlying foraging strategy deployed. Ascertaining whether trap architecture is regulated in response to nutritional history is however notoriously difficult, primarily because little is known about the foraging ecology of trap-building predators^27^.

The practical difficulties faced when attempting to hold all of the confounding variables constant while varying nutritional intake renders application of the geometric framework model to spider webs and silks exceptionally difficult. In the past, researchers have fed spiders a single prey type and manipulated the media on which the prey were reared. For example flies have been reared on nutrient enriched *vs* nutrient poor media to alter their nutritional composition^21^,^25^. However, in this case any changes in feeding strategy of the spider as a consequence of their nutritional history or diet induced variability in the behaviour of the prey remain unquantified. Other researchers have fed spiders a liquefied food source of a pre-determined nutritional composition directly into their mouthparts, thus enabling nutritional history to be controlled^26^,^28^,^29^. However, this method does not simulate a spider’s natural feeding process.

Several studies have demonstrated that the size and architecture of a spider’s web differs with the nutritional history of the spider^24^,^25^,^26^,^28^,^30^. On its own, however this does not demonstrate a link between nutrient intake and web properties because variables other than nutrient intake induces variations in web architectures^7^,^17^,^25^,^28^,^31^. Furthermore, the silks from which the webs are constructed are made of proteins (conventionally called spidroins) and spiders under nutrient stress may need to selectively partition their dietary amino acids between different spidroins and somatic functions^32^. Differential expression of the spidroins by spiders on different diets, which can be identified by a shift in silk amino acid composition, may affect the silk’s tensile properties^26^,^33^. Hence many spider web and silk properties are plastic so may serve as parameters that can be plotted as “performance landscapes” over nutrient space, thus enabling us to ascertain whether these traps are regulated in response to the nutritional history of the foraging spider.

Recently, experiments were done to ascertain whether the giant orb web spider *Nephila pilipes* varied its silk investment and web architectures as a result of extracting different nutrients from crickets or flies when receiving different kinds of vibratory stimuli^30^,^34^. These experiments found that *N. pilipes* extracted different quantities of protein and lipid from the same prey type when different stimuli were applied to their web. Crickets are bigger and more difficult to manipulate than flies so are likely to be handled differently by spiders^7^,^30^,^31^. It thus appears that when the spiders expected to find crickets, as a consequence of detecting cricket-borne vibrations in their webs, they attacked the crickets a certain way, e.g. by wrapping then biting, but when they expected to find flies but encounter crickets they attacked the crickets differently, e.g. by biting then wrapping. Accordingly, they may consume different proportions of the cricket body as a consequence of employing different attack and handling strategies^30^. Whatever the cause of the differential nutrient extraction phenomenon, we exploited it here to induce spiders to extract different quantities of nutrients from a single prey type while controlling their nutritional history. We thenceforth used a geometric framework model to assess whether the spider’s recent nutrient intake was likely to influence its web architecture and silk physico-chemical properties.

## Results

To determine whether the different treatments induced variations in web and silk properties we first compared the final (i.e. those measured on day 21 of the experiment) web architectures, silk tensile properties and amino acid composition and web tension across treatments. Therein we found a significant difference in the web architectures across the three feeding treatments; live crickets (CC), dead crickets with webs stimulated by live flies (CD), or dead crickets without any web stimulation (CO) (Wilk’s *λ* = 0.837, *F*_3,47_ = 3.059, *p* = 0.037). We found that the final catching area (*F*_2,49_ = 7.780, *p* = 0.008) and total silk length (*F*_2,49_ = 8.237, *p* = 0.006) differed significantly across treatments; both of which were greater in the CO feeding treatment than the other two treatments (Supplementary Table S1). The final tensile properties of the major ampullate (MA) silk also differed between feeding treatments (Wilk’s *λ* = 0.752, *F*_3,47_ = 4.371, *p* = 0.04), with ultimate strength and toughness greater in the CD and CO feeding treatments than the CC feeding treatment (Supplementary Table S2). The final silk amino acid compositions differed (Wilk’s *λ* = 0.577, *F*_3,47_ = 2.663, *p* = 0.040) across feeding treatments, with serine (*F*_2,49_ = 3.503, *p* = 0.038) lower in the CD treatment than the other two treatments, and proline (*F*_2,49_ = 3.893, *p* = 0.027) higher in the CD treatment than the other two treatments (Supplementary Table S3), indicating that spidroin expression varied across treatments. Web tension did not significantly differ across treatments (*F*_2,49_ = 1.080, *p* = 0.070).

**Figure 1:**
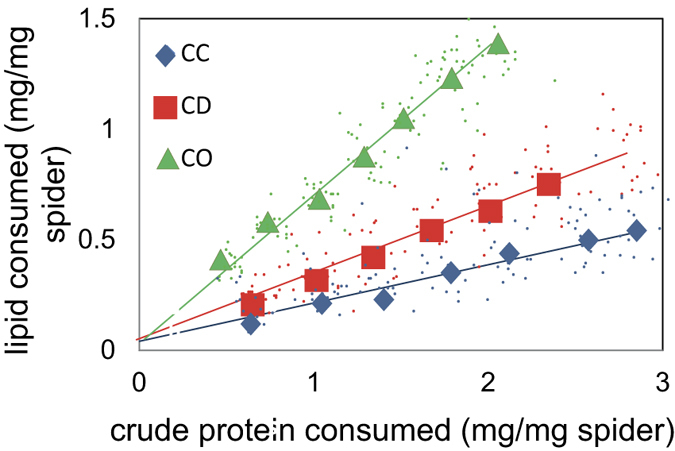
Nutritional rails of mass (mg/mg spider) of crude protein consumed *vs* lipid consumed by *Nephila pilipes* when fed either: live crickets (CC) dead crickets with webs stimulated by live flies (CD), and dead crickets without any web stimulation (CO). The major data points represent the accumulated mean values at each of seven feeding rounds. The minor data points represent the accumulated values of individuals across the seven feeding rounds, which we used to estimate nutrient space for subsequent analyses.

We then measured the accumulated mass of crude protein and lipid extracted from the cricket prey by spiders in the CC, CD and CO treatments and found that they significantly differed (Wilk’s *λ* = 0.752, *F*_3,47_ = 5.155, *p* = 0.004). Nutritional rails for spiders in each treatment were produced by plotting the accumulating mean masses of crude protein *vs* lipid consumed across their first seven feeding rounds (Fig. 1).

**Figure 2:**
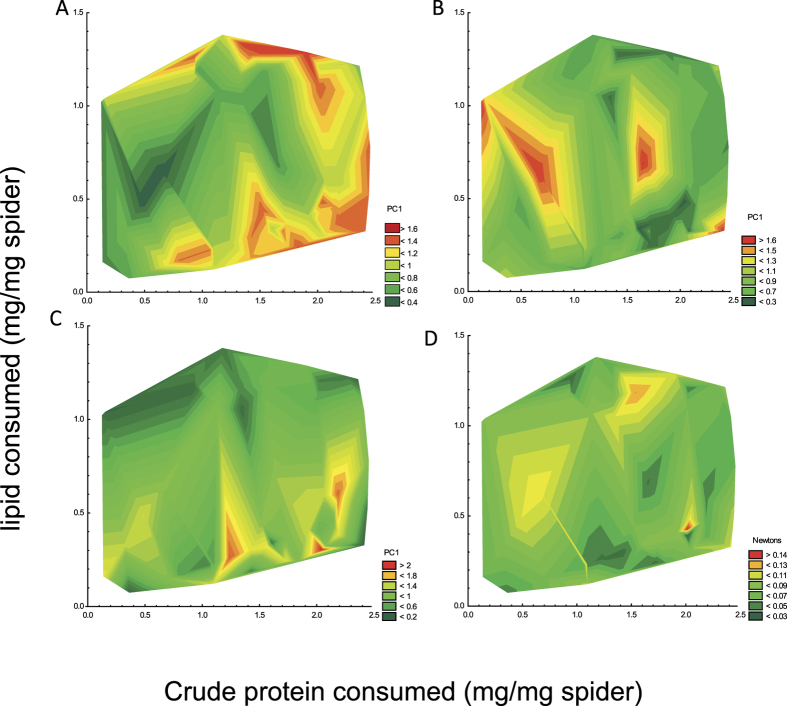
Multivariate response surface or so called “performance landscapes” (Simpson *et al*.^9^), for web architecture (**A**), silk tensile properties (**B**), silk amino acid composition (**C**) and web tension (**D**) across nutrient space. The landscapes were generated by overlaying web architecture, silk property, and silk amino acid composition principal component scores and the directly measured web tension values over nutrient space, which was ascertained from the range of our experimentally derived crude protein *vs* lipid consumption values across treatments. The red-brown shaded areas within each panel represent regions where performance measures are the highest. The green shaded areas representing regions where performance measures are the lowest.

Nutrient space was represented by the entire range of crude protein *vs* lipid consumption values plotted between the three treatments (see Fig. 1). We derived single parameters for web architecture, silk tensile property and silk amino acid composition for each individual at each feeding round and used them, along with the web tension measurements, to overlay performance landscapes over the nutrient space. The red regions of our subsequent performance landscapes (Fig. 2) represented the regions in nutrient space where the performance measures were greatest (the “peaks” in the landscapes) and the dark green regions were those where the performance measures were the lowest (the “troughs” in the landscapes). We found that the web architecture performance landscape peaked when crude protein intake was high and when lipid intake was moderately low to high (Fig. 2A). Contrarily, the silk tensile property performance landscape peaked when crude protein intake was low and lipid intake high, representing what appears to be an inverse relationship with the web architecture landscape, although there was a secondary peak in the silk tensile property performance landscape at intermediate levels of crude protein and lipid intake (Fig. 2B). Silk amino acid composition performance landscapes, which can be construed as representing spidroin expressions, were by and large smooth across nutrient space with three small peaks in the landscape where crude protein intake was high and lipid intake low to moderately low (Fig. 2C). The web tension performance landscapes remained relatively smooth throughout nutrient space, with two peaks evident when lipid intake was high and protein intake high and when lipid intake was high and protein intake moderately low (Fig. 2D). Our generalized additive models suggested that the peaks in the landscapes represented multiple functional maxima for all parameters (Table 1). No single “intake target” within crude protein *vs* lipid nutrient space could thus be identified.

## Discussion

Here we induced the giant orb web spider *Nephila pilipes* to extract different quantities of protein and lipid from a single prey type; laboratory reared crickets. Our measurements of the final web and silk property variations across feeding treatments largely agreed with similar studies^7^,^30^,^34^,^35^,^36^. We then used the web and silk property measurements made after each feeding round to build a geometric framework model. The subsequent web and silk performance landscapes revealed multiple peaks and troughs, which we interpreted as functional maxima and minima for all parameters. More importantly, the locations of the peaks and troughs in the performance landscapes differed across nutrient space suggesting that each of the different web and silk parameters were under different forms of regulation as they moved through nutrient space.

Variations in energy uptake over nutrient space may confound geometric framework models when using protein against lipid intake to construct nutrient space^9^,^11^. The grading patterns of the contours become important in such models as contours in the performance landscape that grade from the origin outward into nutrient space indicate that energy intake has a greater influence of over “performance” than nutrient balance. However, when the contours grade left or right throughout nutrient space they indicate that nutrient balance has a greater effect on “performance”^11^. Since all of the contours in the web and silk performance landscapes that we derived graded to the left or right throughout nutrient space and not from the origin outwards, regardless of the position of the functional maxima, we concluded that nutrient balance and not energy intake was influential over the performance landscapes for the parameters examined herein.

Of the performance landscapes that we plotted, those for web architecture and silk tensile properties had the most prominent peaks and troughs. This suggests that variability in web architecture and silk tensile properties are most influenced by the spider’s recent nutritional intake^30^. We found here that the web architecture performance landscape peaked when lipid and/or crude protein intake was greatest. Since the spiders in the CO treatment group (the group with from which greatest proportion of protein was extracted) invested most heavily in radial threads and web catching area, the peak in the web architecture performance landscape means that protein intake had a positive influence on the investment of radii and spiral threads^25^,^28^,^30^. We thus surmised that the positive relationship with protein intake is most likely a consequence of the spiders partitioning its protein resources between silk and somatic functions^32^. We saw no obvious pattern in our experimental data that might account for the peak in the web architecture performance landscape when lipid intake was greatest. We, thus, deduced that while the spiders may have partitioned energy between silk and somatic functions as lipid intake increased, this partitioning was not explicated by this experiment.

We found, albeit without performing detailed analyses, inverse patterns in the web architecture and silk tensile property performance landscapes. Since a spider web is made of silk it might at face value be expected that any changes in web architecture over nutrient space need to be counter balanced by changes in silk properties if web functionality is to be retained. Nevertheless, our finding of a secondary peak in the silk tensile property performance landscape without a co-existing secondary trough in the web architecture performance landscape suggests that there may not be any such counter balancing. Confounding influences such as variations in energy intake over nutrient space or the influence of other, unmeasured, nutrients might have obfuscated our interpretations of the influences of protein and lipid balance on the shape of the performance landscapes, or rendered the model untrustworthy at the margins of the nutrient space^11^,^37^. If we, however, limit our interpretations of the landscapes to within the reliable interior of nutrient space it would be reasonable to conclude that web architecture and silk tensile properties inversely co-varied across nutrient space, but more experimental information regarding the properties of the silks used in webs of a particular architecture are needed to determine whether web and silk properties do indeed counter balance. The silk amino acid composition and web tension performance landscapes consistently showed little variation across nutrient space, so they did not seem to have any kind of association with any of the other performance parameters. We predicted that while web architectures and silk tensile properties are variable and probably influenced by the spider’s nutritional history, silk amino acid compositions and web tension appear to be more homeostatic.

Our amino acid composition performance landscapes had multiple small peaks. These patterns, which we interpreted as being driven by the expression of different spidroins inducing variations in proline and serine compositions across treatments, did not correspond with any patterns seen in our silk tensile property performance landscapes. The MA silk tensile properties thus seemed to vary over nutrient space independently to spidroin expression. Indeed, recent studies on spider MA silk property plasticity within individual spiders have demonstrated that various biochemical actions during spinning change silk protein structure independent of spidroin expression, so variation in the spidroins present does not necessarily induce variability in the mechanical properties^33^,^38^,^39^,^40^.

Since we measured web tension according to Blamires *et al*.^7^ as the ability of the radial threads to resist elastic deformation when a force is applied, our finding that the web tension performance landscape was relatively smooth across the entirety of nutrient space indicated that the elastic properties of the radial threads varied little. We do not know, however, if the smoothness of the web tension performance landscape is indicative of optimized web functionality across nutrient space. Nor do we know the mechanisms by which spiders are able to retain constant web tensions across nutrient space in light of the considerable variations in the web architecture and silk tensile property landscapes. We predict that web tension was constant across nutrient space to expedite the retention of web functionality and is probably facilitated by an interplay between radial thread molecular structure and the properties and internal strength of the substrate anchors^17^,^41^,^42^,^43^,^44^.

We showed herein variability in the shape of multiple web and silk performance landscapes for the orb web spider *Nephila pilipes*. An assessment of the influence of nutritional history over spider web and silk properties, indeed the properties of any predatory trap, has until now eluded researchers because of inherent logistical difficulties with feeding spiders specific nutrients while keeping other factors controlled. We overcame these difficulties by exploiting *N. pilipes*’ ability to extract different quantities of nutrients from a single prey when the web was stimulated in different ways. This enabled us to use a geometric framework to plot multiple web and silk performance landscapes across protein *vs* lipid nutrient space^9^, from which we found multiple functional maxima.

We concluded that *Nephila pilipes* plastically regulates the chemico-physical properties of its trap in response to its nutritional history. Further exploration is needed to ascertain whether any of the functional maxima and minima corresponded with peaks and troughs in spider fitness. Presenting *N. pilipes* with different nutritional choices, as has been done for other predators^1^,^3^,^4^,^15^, will ascertain whether *N. pilipes* forages to reach positions in nutrient space occupied by the functional maxima. Comparative studies using similar sized spiders from similar habitats that feed on similar prey will provide insights into the evolutionary significance of features such as the multiple peaks in the performance landscapes. Furthermore, the functional consequences of variations across web performance landscapes could be tested by measuring or modelling how webs built under various nutritional constraints dissipate impact forces.

## Methods

### Spider collection and pre-treatment feeding

We collected 60 sub-adult female *N. pilipes* (15–20 mm in body length) from locations near Taichung, Taiwan, and returned them to the laboratory at Tunghai University. We measured their body length to ±0.1 mm and mass to ±0.001 g using digital callipers and an electronic balance, respectively, before placing them in individual 900 × 900 × 330 mm reinforced plywood enclosures with front and back Perspex screens within a greenhouse where they were subject to natural temperature, humidity and dark: light fluctuations. Once all of the spiders had built orb webs (after approximately 4 days) we commenced the pre-treatment feeding of one mealworm daily over 5 days. We re-weighed all of the spiders after the pre-treatment feeding and measured their web architectures and silk chemical and physical properties, as outlined in the “Web Measurements” and “Silk Physico-Chemical Property Measurements” sections below. Spiders that did not feed during pre-treatment and/or spiders that lost >20% of their body weight during the pre-treatment feeding (*n* = 6) were not used in the subsequent experiment.

### Feeding experiment

The 54 spiders used for the following experiment were randomly divided into three feeding treatments (*n* = 18 per treatment): (i) live crickets (CC), (ii) dead crickets with webs stimulated by live flies (CD), or (iii) dead crickets without any web stimulation (CO) using protocols resembling those of Blamires *et al*.^7^,^30^,^34^.

We fed spiders in the CC treatment by a placing live cricket onto their web at a random location. Spiders in the CD treatment had five live flies simultaneously placed onto their web at random locations, which we removed and replaced with one freshly killed cricket immediately prior to the spider attempting to attack the flies. The removal of the flies did not influence the spiders capacity to consume the subsequently placed cricket^7^,^30^,^34^. Spiders in the CO treatment had one freshly killed cricket placed onto their web at a random location. We used laboratory-reared crickets, *Acheta domestica* and house flies, *Musca domestica*, fed carrots and a standard media *ad libitum*. For both the pre-treatment and experimental feeding the spiders were housed in the same plywood enclosures within the same greenhouse.

The day that the first round of feeding commenced was considered day 1 and the first web built by each spider once feeding commenced was considered web 1. Within 12 h of each spider completing each feeding round (indicated by the spider showing no more interest in the remaining prey) we removed the carcasses of the consumed crickets from the web or enclosure, measured the web, collected silks from selected spiders and destroyed the web. All spiders were monitored daily for a new web, upon which another round of feeding commenced. We iterated the experiment for 21 days until its termination. Spiders that failed to build at least 7 webs (one from the CD treatment) or died (two from the CC and one from the CD treatment) within 21 days were not included in the subsequent analyses.

### Nutrient analyses

The carcasses of approximately 30 unconsumed crickets and all cricket carcasses removed from the webs or enclosures during the experiment were dried at 60 °C for 48 h in a drying cabinet (DO45; Deng Yng Instruments, Tainan, Taiwan) and weighed to the nearest 0.001 g using an electronic balance. We extracted lipids from the crickets by soaking them three times in petroleum ether upon which they were vacuum dried and re-weighed. Large crickets (body masses greater than 300 mg) were soaked five times. The quantity of lipids removed was calculated as the difference in the mass of the samples before and after soaking. Nitrogen content was measured by combustion of the weighed vacuum-dried samples in an NC Elemental Analyser (NA2000, Carlo Erba, Rodano, Italy). Crude protein content was calculated by multiplying nitrogen content by 6.25^45^, as previously done in studies investigating predator nutrition^3^,^10^,^46^,^47^. Other forms of insect nitrogen, such as chitin and nucleic acids, may have contributed toward crude protein determination but we did not expect these to be differentially extracted by spiders across our feeding treatments so were considered inconsequential to the outcome of the experiment. The mass of crude protein and lipid consumed by each spider was calculated as the masses of protein and lipid in their prey remains subtracted from the mean protein and lipid masses in whole prey. This value was divided by the mass of the spider to attain a measure of protein and lipid consumption per mass of spider.

### Web architecture measurements

At the completion of pre-feeding and for each subsequent web built over the 21 days of the experiment we removed spiders from their webs and made the following measurements.

We firstly rendered the webs visible by gently spraying them with a mist of tap water and then counted the number of radii and spirals along the four cardinal directions (up, down, left and right), and measured (±0.1 mm) the upper, lower, left and right hub and web radii.

These variables were then used to calculate the following architectural parameters:

Catching area^48^:


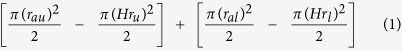


where:


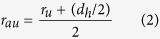



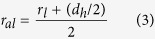


and *r*_*u*_ is the radius of the upper portion of the web, *r*_*l*_ is the radius of lower portion of web, *d*_*h*_ is the width of web, *Hr*_*u*_ is the radius of upper portion of hub and *Hr*_*l*_ is the radius of lower portion of hub.

Total silk length^36^:


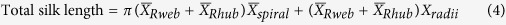


where 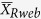 is the average radius of web, 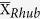 is the average radius of hub, 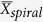 is the average number of spirals and *X*_*radii*_ is the number of radii.

Total spiral length^36^:


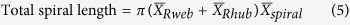


and Mesh height:


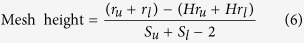


where *S*_*u*_ is the number of spirals in upper half of web and *S*_*l*_ is the number of spirals in the lower half of the web.

### Web tension measurements

We measured web tension, which we defined following Blamires *et al*.^7^ as the ability of the web to resist elastic deformation when a force is applied to the radial threads, on each successive web built by each spider. We collected the webs by placing two circular wooden frames (diameter = 300 mm) with superglue around their rims on either side of a web, ensuring the hub was at approximate the centre of the ring, and moving them toward each other carefully until they touched. The frames were pressed firmly together in order to stick them to each other. Any web components lying outside the frames were burnt away using a stick of hot incense. Since it is the radial threads that enable the web to elastically deform^42^, we laid the frame containing the web flat on a bench and attached a metal s-shaped hook (height = 16 mm) to a radial thread at a point approximately 100 mm from the lower margin of the hub. A microbalance (±0.1 g) was fixed to the movable arm of a dissecting microscope and a ruler was placed vertically perpendicular to the frame. We turned the wheel of the microscope to lift the web to a height of 30 mm. We measured the Newtons force needed (calculated as the mass reading of the microbalance × 9.8) to lift the radial thread. The procedure was repeated four times on different radial threads, all from the lower half of the web, and an average was calculated. All radii were extended less than 20% of their length, i.e. within the functional limits of the silk, thus ensuring we did not deform any other radii in the process. Only the lower half of the orb was used because *N. pilipes* constructs an asymmetric orb so the radii in the upper half of the orb were too short for performing the measurements.

### Silk physico-chemical property measurements

At the completion of pre-feeding and after each feeding round we anaesthetized three randomly selected spiders and taped them ventral side up to a foam rubber platform. We ensured, by noting the allocated number of every spider silked, that we never forcibly silked a spider on two consecutive feeding rounds. Following a 30 minute delay (to ensure that the anaesthesia did not influence the silk properties) we drew silk from the spider’s major ampullate spinnerets using forceps. The extracted silk was taped to a mechanical spool, which was reeled at 5 mms^−1^ for 20 minutes. We viewed the spinnerets under a dissecting microscope during silking to ensure that a single fiber was always spooled.

From each spider five 25 mm sections of taut MA silk fibers were individually mounted onto cardboard frames (open area = 20 × 20 mm, border = 5 mm) with double-sided adhesive tape around its border. A second cardboard frame with double-sided adhesive tape around its border was placed on top of the original and the frames were stuck together securing the silk within by adding one drop of Elmer’s glue (Elmer’s Products, Westerville, OH, USA) at the position where the silk was secured between the frames and squeezing the borders with forceps. The frames containing silk were taped to a microscope slide and examined and photographed under 1000x magnification using a polarized light microscope (BX 50, Olympus, Tokyo, Japan) connected to a UC-series Nikon digital camera. The width of each thread was determined from the photographs using the program Image J (NIH, Bethesda MD, USA). All silks were extracted by the same method by the same researcher (YHT) under controlled temperature (~25 °C) and humidity (~30% R.H.) in still air, so reeling speed and post-spin handling had no influence on the subsequent mechanical properties of the silks.

Tensile tests were performed under controlled temperature and humidity on the frame-mounted silks from each individual approximately 10 days after their collection. We first placed the frames containing single silk fibers within the grips of a UTM Nano Bionix tensile testing machine (MTS Systems Corporation, Oakridge TN, USA), ensuring that the grips held the silk firmly at the edge of the frame. The silks were stretched at a rate of 0.1 mms^−1^ until rupture. The load resolution of the machine was ~2 μN.

Stress (σ) and strain (ε) were calculated by^49^:


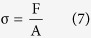


where F is the force applied (load) to the specimen and A is the cross-sectional area of the thread calculated from diameter, assuming a constant thread volume^50^, and


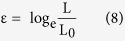


where L is the instantaneous length of the fiber at a given extension value and L_0_ is the original gage length of the fiber. Stress-strain and load-extension curves were plotted for each silk using TestWorks 4.0 (MTS Systems Corporation, Eden Prairie MN, USA), from which we calculated the parameters: (1) ultimate strength; or the stress at rupture, (2) extensibility; or the strain at rupture, (3) toughness; the total work of extension, calculated as the area under the stress strain curve, and (4) stiffness (modulus); the slope of the curve during the initial elastic phase.

The silk remaining from each individual after tensile testing was weighed to the nearest 0.01 mg on an electronic balance and placed into 10 μl tubes (Eppendorf, Hamburg, Germany) and submerged in 99% hexaflouro-isopropanol (500 μl mg^−1^ silk) overnight. The solutions were examined for impurities before being dried and hydrolysed at 115 °C in 6 mol l^−1^ HCl for 24 h. The percentage composition of the amino acids glutamine, serine, proline, glycine and alanine (as they represent ~90% of the total MA silk amino acid composition in most spiders^38^), were determined by reverse-phase high performance liquid chromatography using an Amino Acid Column (Waters Pico-Tag, Milford CA, USA).

### Analyses

To initially determine the effect of treatment on the various web and silk properties, we used repeated-measures (pre-treatment *vs* experimental) MANOVAs and Fisher’s Least Significant Difference post-hoc analyses to compare the final, i.e. those measured on day 21, web architectural parameters (number of radii, catching area, total silk length, total spiral length and mesh height), silk tensile properties (ultimate strength, extensibility, toughness and modulus) and silk glutamine, serine, proline, glycine and alanine compositions between the CC, CD and CO treatments. A repeated-measures ANOVA was used to compare the final web tension values between treatments. Using repeated measures tested for significant differences between pre-treatment and experimental measurements across feeding treatments. All data were non-normally distributed (Kolmogorov-Smirnov tests, *p* < 0.05) so were log_10_ or arcsine (amino acid data) transformed prior to analysis.

We compared the accumulated mass of protein and lipid extracted from the CC, CD and CO treatments by a multivariate analysis of variance (MANOVA) and a Fisher’s Least Significant Difference post-hoc analysis. We plotted the accumulating mean masses of crude protein consumed against the accumulating masses of lipid consumed by spiders across the first seven feeding rounds to construct nutritional rails representing the ratio of the nutrients consumed by the spiders in each treatment^8^.

We used the entire range of values for crude protein consumed and lipid consumed across the three treatments to represent the available nutrient space. To construct the response surfaces of our “fitness landscapes” we calculated first principal component scores derived from correlation matrices to collapse the web architecture (generated from number of radii, catching area, total silk length, total spiral length and mesh height values), silk tensile properties (ultimate strength, extensibility, toughness and stiffness) and silk amino acid composition (glutamine, serine, proline, glycine and alanine composition) measurements for individual spiders at each feeding round to single values in nutrient space. We then generated thin-plate spline contour plots^51^ to map the performance landscapes for the web architecture, silk tensile properties and silk amino acid composition points within nutrient space as well as the directly measured web tension values within nutrient space. We ascertained whether peaks in the landscapes represented functional maxima or were a product of co-varying trends along both axes by independently examining the influences of the predictor variables protein consumed (X variable) and lipid consumed (Y variable) on the response variables: (i) web architecture, (ii) silk tensile properties, (iii) silk amino acid composition, and (iv) web tension, against the influences of X × Y interactions using a quasi-likelihood generalized additive model (GAM)^52^. The degrees of freedom were set at 4 and we ran 15 iterated smoothings of the data.

## Additional Information

**How to cite this article**: Blamires, S. J. *et al*. Spider web and silk performance landscapes across nutrient space. *Sci. Rep*. **6**, 26383; doi: 10.1038/srep26383 (2016).

## Supplementary Material

Supplementary Information

## Figures and Tables

**Table 1:** Results of quasi-likelihood generalized additive models (GAMs) examining the influences of the predictor variables protein consumed (X) and lipid consumed (Y) on variation in the response variables: web architecture, silk tensile properties, silk amino acid composition, and web tension against the influence of X × Y interactions.

Response variables	df	df residual	Predictor variables						Final deviance
			X (lipid consumed)		Y (protein consumed)		X × Y interaction		
			F-ratio	P	F-ratio	P	F-ratio	P	
Web architecture	4	48.024	1.013	0.318	0.026	0.870	24.801	<0.001	2498.320
Silk tensile properties	4	47.945	0.002	0.961	0.481	0.409	36.436	<0.001	1231.432
Silk amino acid composition	4	44.997	0.732	0.396	0.163	0.688	17.310	<0.001	295.051
Web tension	4	48.004	0.034	0.853	0.399	0.530	30.992	<0.001	154.036

Degrees of freedom was set at 4 and we ran 15 iterated smoothings of the data.
