# Supplementary material for: Spider web and silk performance landscapes across nutrient space
Source: Sci Rep. 2016 May 24;6:26383. doi: 10.1038/srep26383 (PMC4877650; doi:10.1038/srep26383)
Supplement: Supplementary Information [file srep26383-s1.pdf]

Spider web and silk performance landscapes across nutrient space

Sean J. Blamires[1,2*], Yi-Hsuan Tseng[3], Chung-Lin Wu[4], Søren Toft[5], David Raubenheimer[6] and I-Min Tso[1,3]

[1]Department of Life Science, Tunghai University, Taichung 40704, Taiwan

[2]Evolution & Ecology Research Centre, School of Biological, Earth & Environmental Sciences, The University of New South Wales, Sydney 2052, Australia

[3]Department of Life Science, National Chung-Hsing University, Taichung 40227, Taiwan

[4]Center for Measurement Standards, Industrial Technology Research Institute, Hsinchu 30011, Taiwan

[5]Department of BioScience, Building 1540, Aarhus University, Ny Munkegade 116, DK-Aarhus 8000 C**,** Denmark

[6]The Charles Perkins Centre, Faculty of Veterinary Science & School of Biological Sciences, The University of Sydney, Sydney NSW 2006, Australia

Supplementary Information Table S1. Mean ± s.e. (Fisher's Least Significant Difference post-hoc analysis: a>b) web architecture parameters for *Nephila pilipes* webs built on day 21 of the experiment across the three feeding treatments: live crickets (CC), dead crickets with webs stimulated by live flies (CD), and dead crickets without any web stimulation (CO).

	Feeding treatment		
Parameters	CC	CD	CO
Number of radii	73.98 ± 29.09	70.43 ± 33.45	76.73 ± 19.88
Web catching area (m^2)	24.52 ± 8.77[b]	26.54 ± 10.69[b]	33.28 ± 8.48[a]
Total silk length (m)	83.67 ± 26.74 [b]	82.85 ± 27.35 [b]	89.36 ± 19.88 [a]
Total spiral length (m)	16.38 ± 2.86	16.90 ± 1.52	17.51 ± 1.94
Mesh height (m)	16.38 ± 2.86	16.90 ± 1.52	17.51 ± 1.94

Supplementary Information Table S2. Mean ± s.e. (Fisher's Least Significant Difference post-hoc analysis: a>b) silk tensile property parameters: ultimate strength, extensibility, toughness, Young's modulus, ultimate tension, and breaking energy for *Nephila pilipes* silks of silks collected on day 21 of the experiment across the three feeding treatments: live crickets (CC), dead crickets with webs stimulated by live flies (CD), and dead crickets without any web stimulation (CO).

	Feeding treatment		
Parameters	CC	CD	CO
Ultimate strength (MPa)	501.169 ± 89.192[b]	621.823 ± 108.721[a]	599.209 ± 65.225[a]
Extensibility (mm mm^{-1})	0.329 ± 0.094	0.385 ± 0.185	0.363 ± 0.066
Toughness (MJ mm^{-3})	109.021 ± 5.291[b]	124.421 ± 7.266[a]	131.671 ± 10.025[a]
Stiffness (GPa)	8.574 ± 0.753	8.857 ± 0.487	8.886 ± 0.275

Supplementary Information Table S3. Mean ± s.e. (Fisher's Least Significant Difference post-hoc analysis: a>b) % amino acid compositions for *Nephila pilipes* silks collected on 21 of the experiment across the three feeding treatments: live crickets (CC), dead crickets with webs stimulated by live flies (CD), and dead crickets without any web stimulation (CO).

Amino acids (% compositions)	Feeding treatment		
	CC	CD	CO
Glutamine	6.23 ± 2.51	7.15 ± 2.47	5.34 ± 2.09
Serine	4.66 ± 0.98 [a]	3.98 ± 0.41 [b]	4.41 ± 0.29 [a]
Proline	5.033 ± 2.74 [b]	6.73 ± 2.08 [a]	4.94 ± 2.72 [b]
Alanine	27.80 ± 1.88	24.86 ± 2.15	26.34 ± 1.87
Glycine	39.49 ± 12.81	42.91 ± 4.24	43.75 ± 1.73
